# Laparoscopic management of spontaneous vaginal vault dehiscence and bowel evisceration 17 years following total abdominal hysterectomy

**DOI:** 10.1186/s10397-017-1004-6

**Published:** 2017-04-11

**Authors:** Georgina Baines, Simon R. Jackson, Natalia Price

**Affiliations:** Oxford University Hospitals Trust, John Radcliffe Hospital, Headley Way, Oxford, OX3 9DU UK

**Keywords:** Hysterectomy, Complication, Vault, Dehiscence, Laparoscopy

## Short communication

We present a case that demonstrates the use of combined vaginal and laparoscopic approach in two stages to repair a late complication of hysterectomy.

A 55-year-old woman presented 17 years following a total abdominal hysterectomy and bilateral salpingo-oophrectomy for severe endometriosis. She was fit and well with a BMI of 21.

Presenting complaint was a 2-day history of frank haematuria. On examination, the only finding of note was a small tear and some blood at the vaginal vault.

On defecating 12 h later, loops of bowel protruded through the vagina and she presented to the gynaecology unit. On arrival, she was haemodynamically stable. A decision was made to examine under anaesthesia, and broad spectrum antibiotics were started.

In theatre, under anaesthesia, the small bowel was reduced in Trendelenburg. A 2.5-cm defect at the apex of the vault was noted with some dusky, non-viable tissue near the thinned edges. The margins of the vault defect were trimmed and repaired horizontally in two layers (polyglactin, interrupted). Laparoscopic examination of the small bowel showed hyperaemic, viable intestine and complete repair of the vault defect. She recovered well. Histopathology of the vault specimen revealed atrophic vaginal epithelium with fibrosis, epidermal inclusion cysts and calcification.

After 6 weeks, the examination revealed a well-healed vaginal vault. There was a long discussion about the possibility of apical suspension of the vaginal vault to reduce mechanical stress and prevent recurrence.

The patient decided to proceed with laparoscopic sacrocolpopexy at 16 weeks following primary closure.

Laparoscopy revealed thin vaginal mucosa in spite of intense topical oestrogen therapy. The bladder and rectum were reflected from the vagina. A Y-shaped type 1 light weight polypropylene mesh was placed over and sutured to the upper half of anterior vaginal wall and two thirds of posterior wall of the vagina with interrupted polydioxanone sutures. Great care was taken to keep the mesh lying flat over vaginal mucosa to minimise erosion. The vault was suspended from the sacral promontory by the tail of the Y-mesh using Protak fasteners [1], and complete mesh peritonisation was performed with poliglecaprone. Continuous topical oestrogen therapy was re-commenced 2 weeks postoperatively.

She made a good recovery. At 3- and 6-month postoperative follow-up, there was no evidence of mesh erosion. Vaginal mucosa looked healthy with no prolapse and the patient was symptom free.

Vaginal vault dehiscence after hysterectomy is defined as the separation of the cut edges of the vaginal incision, with or without the herniation of the pelvic or abdominal contents [2].

It has been reported from 6 weeks to 1.6 years [2] to a maximum of 30 years following hysterectomy [3].

Systemic risk factors for cuff dehiscence include age, increased abdominal pressure (chronic cough or heavy lifting) and factors affecting healing such as steroid use. Local risk factors include vaginal atrophy, haematoma, endometriosis and infection [4]. In the case discussed, absence of hormonal replacement (approximately 15 years) would have contributed. A recurrence of endometriosis at the vault could also weaken the tissues [5].

The defect can be repaired abdominally, vaginally, laparoscopically or a combination of two. Selection depends on many factors. In this case, the initial laparoscopy provided an opportunity to ensure there was no visceral damage and assess complete closure internally [6].

There have been procedures described which provide integrity to the repaired defect. Here, the secondary laparoscopic sacrocolpopexy aims to provide vaginal reinforcement and support, preventing recurrence.

In this case, we propose that a good outcome is achieved by a combined technique of vaginal and laparoscopic approach along with a secondary procedure to correct and support the vault.
